# Editorial: Borderline Personality Disorders in Adolescents: Clinical Challenges and Recent Advances

**DOI:** 10.3389/fpsyt.2022.854833

**Published:** 2022-02-17

**Authors:** Silvio Bellino, Paola Bozzatello

**Affiliations:** Department of Neuroscience, University of Turin, Turin, Italy

**Keywords:** borderline personality disorder, adolescence, diagnosis, treatment, trauma, temperament, genes, environment

Early onset of Borderline Personality Disorder (BPD) in adolescence is a topic that has obtained growing interest in recent years. Nevertheless, validated procedures for early identification and precocious therapeutic interventions are still lacking in this population.

Clinical studies on this topic recommended that both risk and protective factors and the complex and mutual interactions of temperamental traits, attachment style, adverse childhood experiences, and neurobiological abnormalities, should be considered (1–5). In addition, a careful examination of the role of each individual which is part of patient's life (parents, teachers, health providers) is required in order to improve the approach to detection and management of BPD in young age (6). The contribution of relational and environmental factors, particularly traumatic events (emotional neglect, physical, and sexual abuse), to the onset of BPD in adolescence and early adulthood has been evaluated in several studies, with an increasing interest for the reciprocal influences with the effects of genes and temperament. A further issue to be addressed in this field of research concerns the key role of BPD in early age as a predisposing condition for other psychiatric disorders with high incidence in adolescence, such as mood disorders, anxiety, substance abuse, eating disorders, obsessive-compulsive disorder, non-affective psychosis, and conditions with prominent somatic symptoms (7).

In the present issue of Frontiers in Psychiatry, Baptista et al. focused their investigation on the cognitive, ecological, and developmental basis of self-disturbance in BPD adolescents. When characterizing self-disturbance, is important to evaluate the sense of agency and to put it in relation with early adverse life events. The sense of agency of BPD individuals is particularly influenced by external information and BPD development can be considered as the result of early experiences of adversity that threaten the patients' survival because of their hypersensitivity to environmental stimuli. Life-history theory, a model of evolutionary developmental biology, is proposed by Authors as a relevant framework to explain how agency-based symptoms emerge, as a biased locus of control or abnormal self-efficacy. This approach considers implications for the prevention and management of the disorder and suggests that current psychosocial interventions might be more effective with a focus on global health.

Bozzatello et al. reviewed studies published on PubMed in the last 20 years to estimate how different types of childhood trauma (sexual, physical abuse, bullying, and neglect) can increase the risk of developing BPD and shape its evolution when symptoms emerge during adolescence. Patients with BPD report childhood abuse and other traumatic experiences more frequently than patients with other personality disorders and the interplay of temperamental traits of impulsiveness and negative affectivity, environmental, and genetic factors with early traumatic experiences can promote onset of BPD in young age. It is also true that adverse childhood experiences are associated with functional and volumetric brain abnormalities, involving gray matter volume, white matter connectivity, neurotransmission mechanisms, function of HPA axis, and endogenous opioid system.

Guénolé et al. investigated the relationship in BPD female adolescents between non-suicidal self-injuring (NSSI), another core feature of young patients with BPD, and the Sidney Blatt two-polarities model of personality development, centered on the psychological processes of interpersonal relationship and self-definition. The model indicates that NSSI represent a way of coping affliction originating from interpersonal troubles and seems to be related with severity of BPD symptoms. Borderline Personality Disorder patients obtained significantly higher scores than healthy controls on the two Depressive Experience Questionnaire (DEQ) factors of Neediness and Self-criticism and significantly lower scores on Self-efficacy. Results show that the factor Neediness of the DEQ is a significant predictor of the presence of NSSI in BPD adolescents. So, patients with immature interpersonal relatedness should be monitored in clinical practice because of the high risk to self-injuring behaviors.

Robin et al. evaluated the role played by parental distress, history of stressful life events (SLEs), attachment, alexithymia, and hopelessness on the affective symptoms of BPD during adolescence. Borderline Personality Disorder adolescents often report emotional dysregulation, insecure attachment, a history of SLEs, and dysfunctional parent–child interactions. The study highlights the contribution of altered attachment, independently from the environment, in development of emotional dysregulation. An interesting finding of the study is that accumulation of SLEs decreases the sense of hopelessness and affective symptoms. Authors conclude that BPD patients show emotional reactions with two identified pathways: the first involving attachment and the emotional dysregulation process for parent–child interactions; the second concerning the interaction of SLEs and alexithymia. Unexpectedly, in insecure conditions, repeated adversities can generate paradoxical effects, including a minor expression of affective symptoms and hopelessness.

Finally, Begin et al. identified profiles of risky sexual behaviors (RSB) profiles in adolescents and young adult subjects related to development of BPD psychopathology. Three different RSB profiles were identified: low RSB profile (77.7%); unprotected sex in relationship profile (13.3%), and impulsive sex outside relationship profile. The last profile is more often associated to BPD characteristics. These findings highlight the difficulties of BPD patients to integrate aspects of sexuality, intimacy, fidelity, and love.

The contributions to this special issue on BPD in adolescence provide the following conclusions:

- BPD development can be considered as the result of interactions between early adversities and hypersensitivity to external information, with relevant implications for prevention and management of the disorder;
- the interplay of temperamental traits, environment, genes, and neurobiological factors with early traumatic experiences promote early BPD onset;
- psychopathological models of early BPD development suggest that non-suicidal self-injuries can be a way of coping affliction that origins from interpersonal troubles and are related with severity of BPD symptoms;
- the RSB profile indicated as “impulsive sex outside relationship” is characteristic of BPD in adolescence.

## Author Contributions

All authors listed have made a substantial, direct, and intellectual contribution to the work and approved it for publication.

## Conflict of Interest

The authors declare that the research was conducted in the absence of any commercial or financial relationships that could be construed as a potential conflict of interest.

## Publisher's Note

All claims expressed in this article are solely those of the authors and do not necessarily represent those of their affiliated organizations, or those of the publisher, the editors and the reviewers. Any product that may be evaluated in this article, or claim that may be made by its manufacturer, is not guaranteed or endorsed by the publisher.
